# Modelling the reopen strategy from dynamic zero-COVID in China considering the sequela and reinfection

**DOI:** 10.1038/s41598-023-34207-7

**Published:** 2023-05-05

**Authors:** Sijin Wu, Zhejun Huang, Susan Grant-Muller, Dongfeng Gu, Lili Yang

**Affiliations:** 1 Department of Statistics and Data Science, Southern University of Science and Technology, Shenzhen, 518055 China; 2 Institute for Transport Studies, University of Leeds, Leeds, LS2 9JT UK; 3 School of Public Health and Emergency Management, Southern University of Science and Technology, Shenzhen, 518055 China

**Keywords:** Environmental social sciences, Mathematics and computing, Computational biology and bioinformatics

## Abstract

Although the dynamic zero-COVID policy has effectively controlled virus spread in China, China has to face challenges in balancing social-economic burdens, vaccine protection, and the management of long COVID symptoms. This study proposed a fine-grained agent-based model to simulate various strategies for transitioning from a dynamic zero-COVID policy with a case study in Shenzhen. The results indicate that a gradual transition, maintaining some restrictions, can mitigate infection outbreaks. However, the severity and duration of epidemics vary based on the strictness of the measures. In contrast, a more direct transition to reopening may lead to rapid herd immunity but necessitate preparedness for potential sequelae and reinfections. Policymakers should assess healthcare capacity for severe cases and potential long-COVID symptoms and determine the most suitable approach tailored to local conditions.

## Introduction

The COVID-19 pandemic has profoundly affected public health, economies, and societies worldwide. China, one of the first countries to face the outbreak, consistently adopted the dynamic zero-COVID policy, characterized by strict control measures, mass testing, quarantine, and targeted lockdowns. While this approach has effectively controlled the spread of the coronavirus within China's borders, it is constrained by public health capacity and many social-economic burdens. In addition, as the global landscape evolves and other countries adopt various reopening strategies, it becomes essential for China to consider suitable reopening strategies. Hence, the transitions from strict restrictions to reopening still have valuable insights and lessons to study.

Moreover, the value and advantages of China's reopening become particularly significant when considering the effects of sequela and long COVID on public health systems. Previous studies on China's COVID-19 situation did not extensively address reinfection and long COVID symptoms, as there were insufficient cases available to support related research before reopening. Nevertheless, experiences from other countries indicate that implementing reopening policies can lead to massive waves of infection and subsequent issues^1^, such as increased mortality rates due to reinfection^2^, public health strains arising from COVID-19 sequelae^3^, and long-term socio-economic impacts resulting from labor shortages^4^. Therefore, we assert that assessing the challenges posed by a large population of individuals with sequelae conditions is crucial following the implementation of reopening policies. This approach enables healthcare providers to allocate appropriate resources and draw on the experiences of countries that have already reopened, facilitating the management and treatment of patients with sequela and long COVID. In turn, this leads to improved health outcomes and reduced long-term complications.

Our study aims to discuss reopening strategies for China that consider these factors in evaluating different scenarios and potential outcomes. The modeling methods play critical roles in discussing and simulating the effects of public health interventions. There are broadly two modeling methods to affect the epidemic process incorporated by policy intervention, compartment models and agent-based models (a.k.a. individual-based models or microscopic models). A compartmental model uses a dynamic system of differential equations to describe the changes in infectious status among the population. Due to easy-to-use and convenient calculation properties, compartmental models have been widely used to simulate the COVID-19 epidemic trends in situations with various interventions. Rosanna et al. employed the region and age-stratified compartmental model considering the vaccine and restriction policies for depicting the dynamics of Omicron epdemic^5^. Levin’s group proposed a network-based SEIR model with short-time travel exposure by infections to predict the spread of COVID-19 in Minnesota^6^. Wu et al. imported the age-structured SEIR model to find the optimal vaccine distribution policy^7^. Cai et al. considered age-specific vaccine coverage, vaccine efficacy, waning of immunity, antiviral therapies and NPIs, and proposed an age-structured stochastic susceptible-latent-infectious-removed-susceptible model to project COVID-19 burdens under hypothetic mitigation scenarios in Shanghai^8^. However, compartment models have their drawbacks. First, they usually assume homogeneous mixing among the subpopulation, which means that the transmission or other behaviours have a certain degree of homogeneity^9,10^. Second, due to the complexity of real-world human behaviours and policy interventions, they are hard to depict detailed interactions without considering individual features. Also, the natural transfer in China from Dynamic Zero-COVID policy to reopen relies on the detailed description of the policy implementation, which usually aims at individual-level containment, for example, quarantining the household members and possible colleagues of detected infectious individuals. Hence, the current study adopted the fine-grained agent-based model to approximate how the virus spreads in complex social networks and against various policy interventions.

In this study, Shenzhen acts as the case study city, a major sub-provincial city with 17.56 million citizens bordering Hong Kong. As one of China's fastest-growing cities, it has a high population density. Shenzhen presents unique challenges for implementing COVID-19 control measures, such as physical distancing and contact tracing. Studying the city's experience can provide valuable insights into how to address these challenges in similarly dense urban settings. This makes it an ideal case study for understanding how COVID-19 control measures might be implemented in a dynamic and growing urban environment. Furthermore, as a leading technology hub, Shenzhen has access to information-based infrastructures to support COVID-19 control measures. In 2022, Shenzhen’s restrictions were characterized by frequent city-level routine tests and sensitive community lockdown thresholds (where a small number of detected cases result in mobility restrictions for all community members).

So, we first simulate the effects of varying the lockdown thresholds from ten to one thousand new cases while maintaining frequent routine testing. We then assess the sustainability of these control measures by analyzing the simulation results in terms of daily infections and severe cases.

Next, we simulate four reopening strategies that transition from Dynamic Zero-COVID, characterized by removing lockdowns and reducing the frequency of routine testing and quarantine. In contrast to Shenzhen's current policy, which requires a 24-h COVID-negative passport for citizens, we consider the following four scenarios:

- Retain the 48-h routine test at the city level;
- Retain the 7-day routine test at the city level;
- Retain the 14-day routine test at the city level;
- Eliminate routine testing and quarantine requirements.

In analyzing these four simulated scenarios, we focus on two key metrics: the number of severe cases requiring public healthcare resources (such as ICU) and the number of long-COVID conditions. We emphasize these metrics because, when implementing any reopening strategy, the death rates and associated public health issues are more uncertain and crucial to monitor than daily infections.

Lastly, based on the simulation outcomes, we aim to offer recommendations and insights to help public healthcare systems prepare for the upcoming challenges in the conclusion section of the study.

## Results

**Figure 1:**
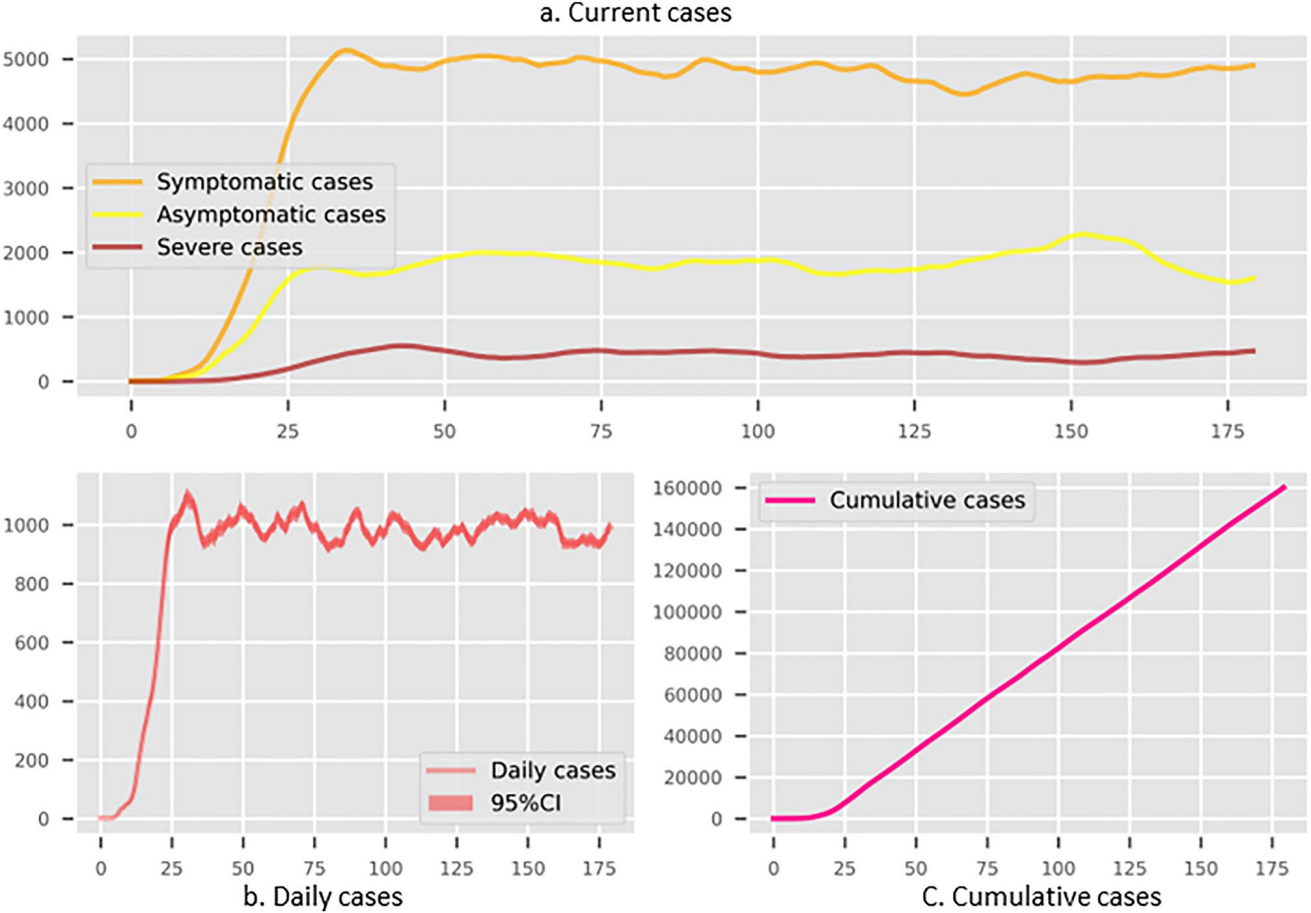
The simulated results for Shenzhen with non-compulsory test and self-isolation with lockdown threshold as 10 per community. (**a**) The existing infections in different stages. (**b**) The daily new cases. (**c**) The cumulative infected cases. Three subplots have the common x-axis representing the number of simulation days.

**Figure 2:**
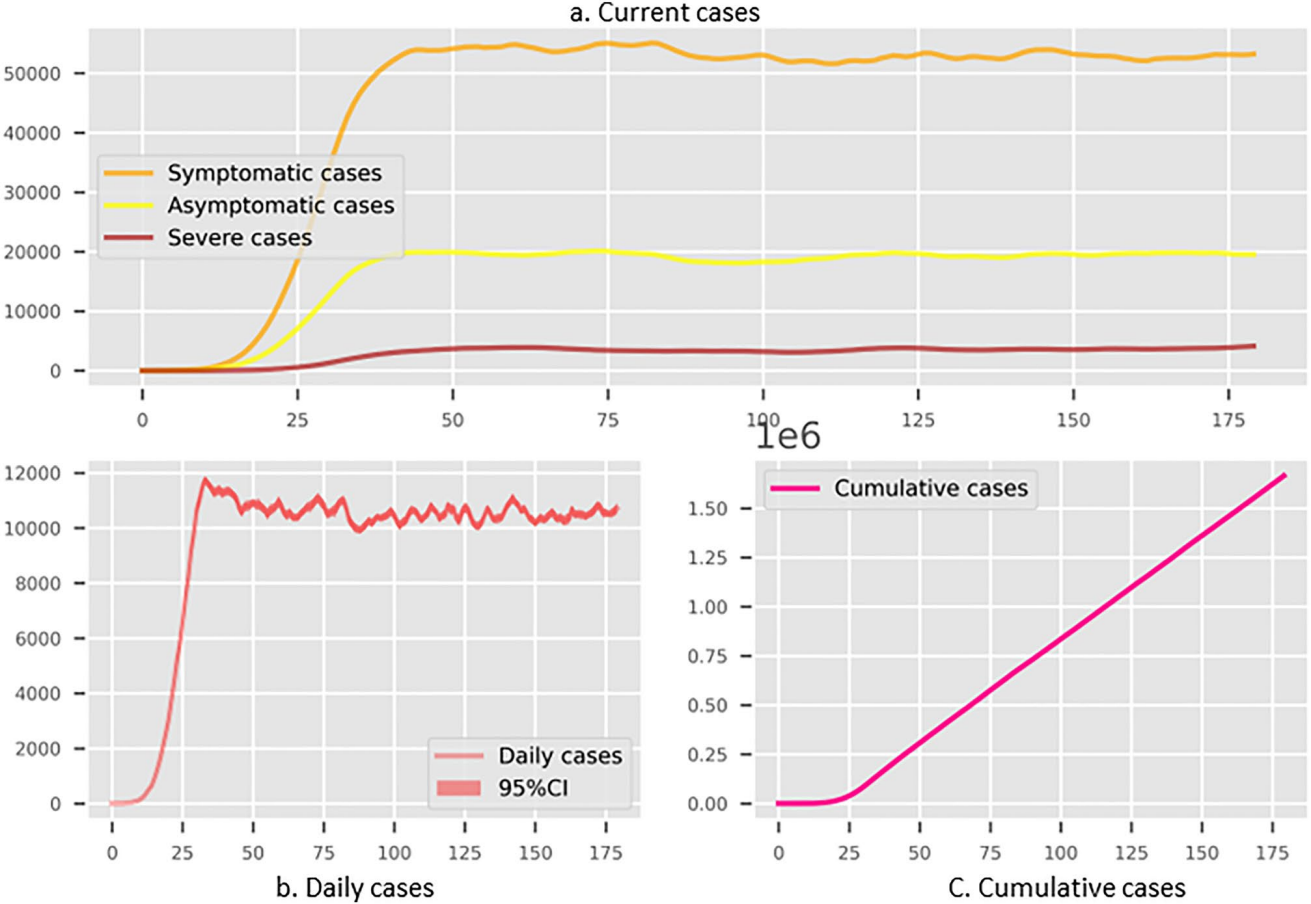
The simulated results for Shenzhen with non-compulsory test and self-isolation with lockdown threshold as 100 per community. (**a**) The existing infections in different stages. (**b**) The daily new cases. (**c**) The cumulative infected cases. Three subplots have the common x-axis representing the number of simulation days.

**Figure 3:**
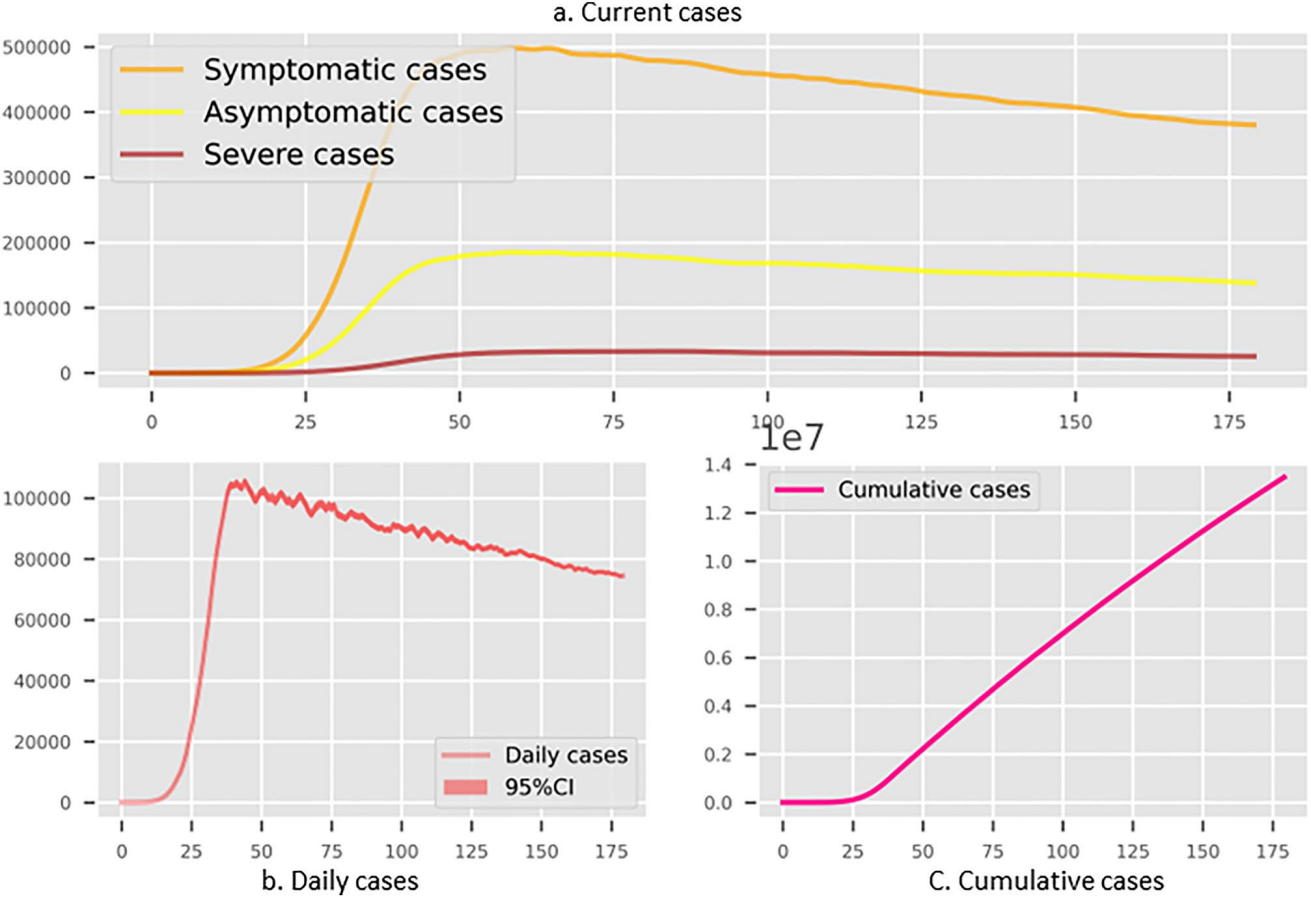
The simulated results for Shenzhen with non-compulsory test and self-isolation with lockdown threshold as 1000 per community. (**a**) The existing infections in different stages. (**b**) The daily new cases. (**c**) The cumulative infected cases. Three subplots have the common x-axis representing the number of simulation days.

The reopening scenarios and consequences of taking Shenzhen as the case study are displayed here. The initialisation of the epidemic is the Omicron era, and only a few cases are detected at the beginning stage in Shenzhen, whose specific hyper-parameter settings are presented in S3. The discussed the Dynamic Zero-COVID policy consists of three options: city-level routine test, quarantine of the related individuals, and lockdown if the situation exceeds the decision-makers threshold. The natural way of transferring the Dynamic Zero-COVID policy is to release the lockdown thresholds, which means the few cases in the community are tolerant, and other community members could maintain normal mobility. For example, we set the lockdown threshold values, 10 and 1000 infections, in a typical living community with around 30,000 residents separately to compare the trends of epidemic development. Meanwhile, the regular test frequency is as same as before. A 48-h test, which means the daily policy efficiency is 0.5, is required for each resident. The complete simulation results are displayed in Figs. 1, 2 and 3. The subplots of each figure display simulation results from the existing cases in different conditions, the daily new infections and cumulative infections. The results show that under regular control measures and lockdown policies, the epidemic will form a fluctuating trend, and its frequency and duration will change with the lockdown threshold. If the lockdown threshold is high, the epidemic recurrence time will be short; if the lockdown threshold is low, the epidemic recurrence duration will be extended. On the other hand, the value of the lockdown threshold affects the upper limit of the number of new cases. Figure 1 demonstrates that the small lockdown threshold, 10, for each community could constrain the rise of current cases and keep them at a low level. However, it would require much longer to achieve the herd immunity shown in Figs. 2 and 3. On the other hand, a loose standard to restrict the community could cause amounts of daily cases, and there might be existing hundreds of thousands of infections that might lead to public panic and health burdens. The positive results are that the peak values of daily and existing cases will drop after 50 days.

**Figure 4:**
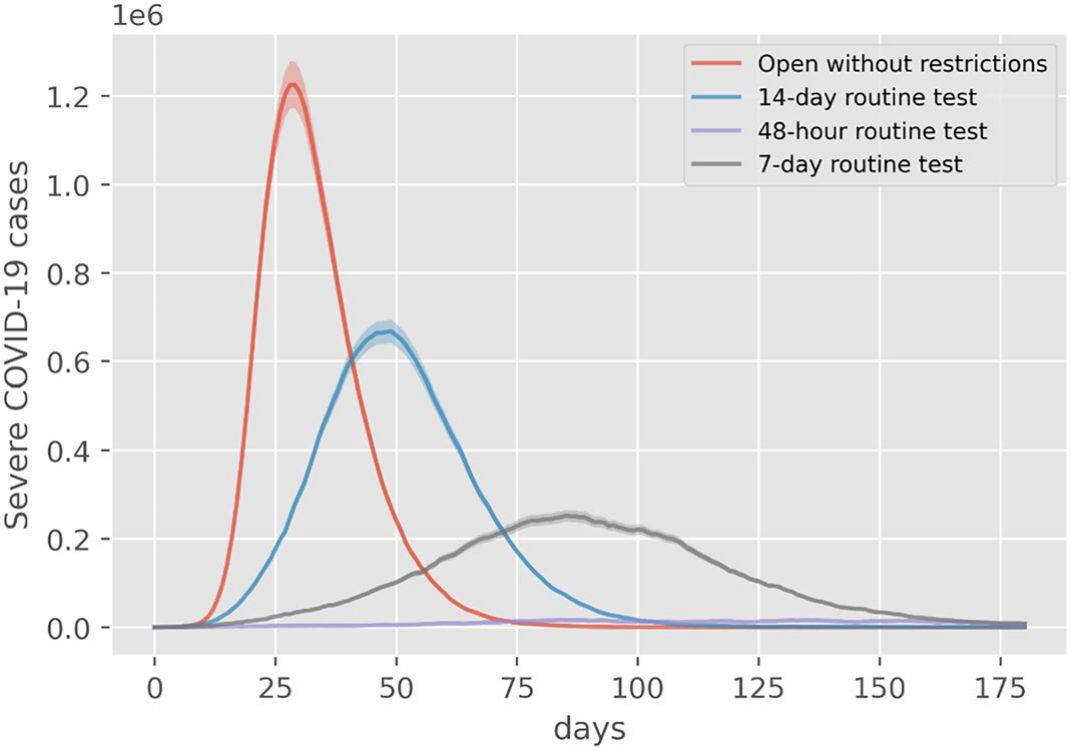
Severe cases under different reopen strategies.

**Figure 5:**
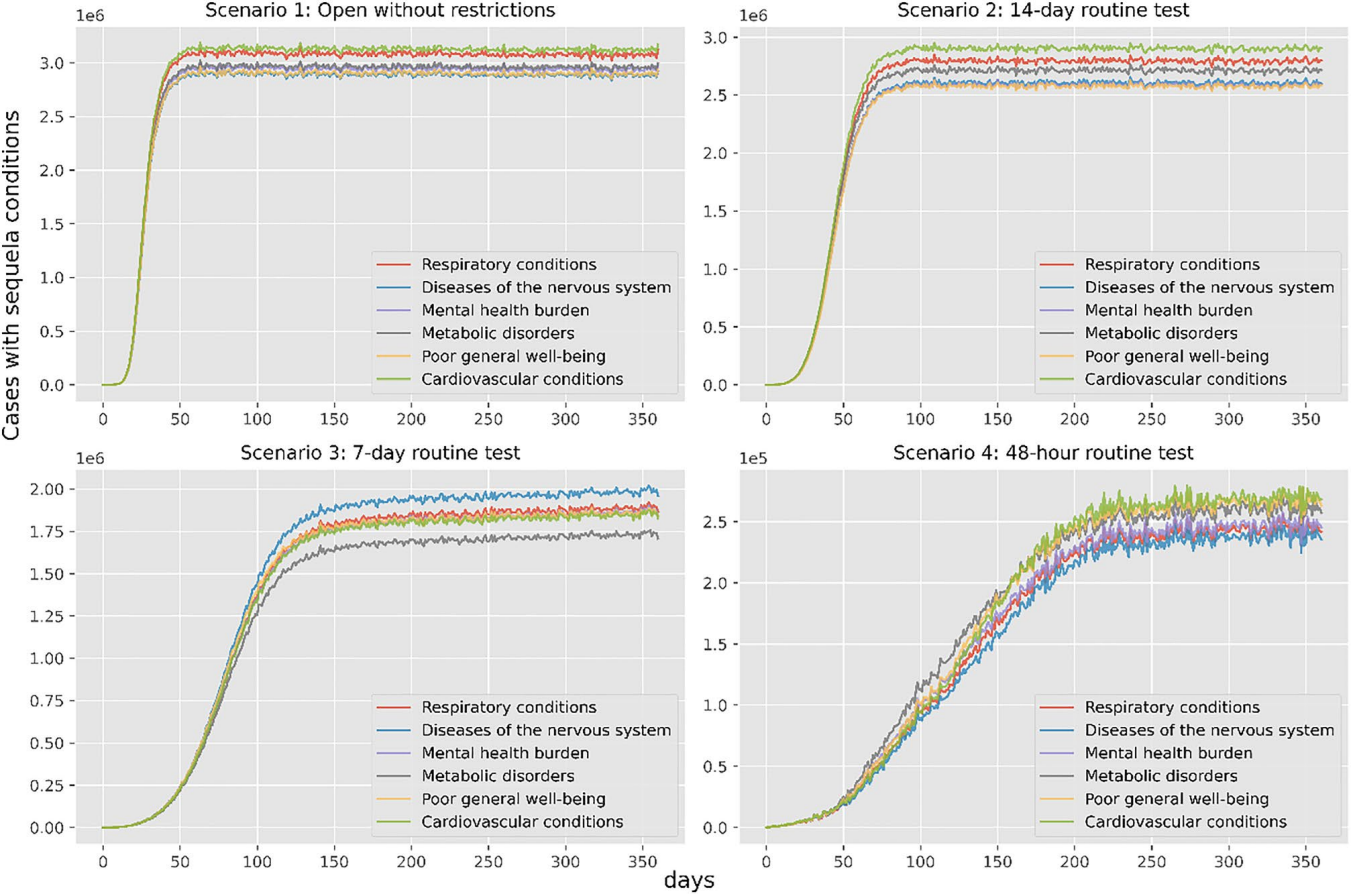
Estimated long-covid cases having different symptoms under four reopen strategies.

Secondly, the study simulates the reopening of Dynamic Zero-COVID by non-use of lockdown while keeping routine tests and quarantine in different frequencies. And the simulation results will be addressed the severe cases and sequela symptoms as the policy focus changes. Figure 4 displays the changes in severe cases simulated under four reopen strategies implemented in Shenzhen. From Fig. 4, the general pandemic pattern without lockdowns is that more strict routine tests and quarantines could shorten the peak value and delay the peak time of severe cases, which implies that the local decision-makers could determine the frequency of routine tests according to the capacity of incoming severe cases simulated. Also, decision-makers should consider the consequences of reopening policy regarding the long COVID conditions. From Fig. 5, the simulated long COVID conditions with different sequelae review that if the reopen strategy is too urgent, many long COVID issues will soon appear. Also, the simulation results about reopening strategies point out that decision-makers should prepare well for the cascading consequences of how fast to release control measures. For example, there might be an outbreak of sequela in Shenzhen after the first wave of infections. At that time, the burdens of the public healthcare system might be transferred from curing COVID conditions to the Long COVID conditions. The corresponding systems and experts, like psychologists, could lead public attitudes toward active mental health in advance.

## Discussions

Reassessing COVID-19 reopening policies is of paramount importance for the ongoing management of coroonavirus spread and safeguarding public health in China. This study scrutinized the background of public policy regarding COVID-19 in China, emphasizing the significance of considering post-implementation outcomes, including long COVID symptoms and reinfections. To this end, we employed a fine-grained agent-based model in Shenzhen, simulating a variety of reopening scenarios. In future research, we plan to compare different regions and find a customized strategy considering their heterogeneous demographic factors in urban systems^11^, such as the temperature and urban recycling systems^12^.

As a mild transition from a dynamic zero-covid policy, increasing the tolerance of infected cases to lockdown is a buffered way. The former policy adopted strict control measures that few cases detected would lead to a community-level lockdown. Hence, we try to simulate what would happen if the lockdown threshold values are released to 10, 100, and 1000 for a typical community with about 30,000 residents. Meanwhile, the routine COVID test and following quarantine measures are kept. From the simulation results, the peak values of infections are determined by the threshold values. In other words, the lockdown measures could maintain infections and limit the virus spread. In addition, the number of severe cases could be kept at a stable level, making it possible to sustain the healthcare system. However, this will lead to a heavy price to maintain the dynamic zero-COVID policy. As the infection level is controlled, the epidemic will last longer, especially for the small lockdown thresholds. There could be other potential economic recessions such as loss of income, increased unemployment, and reduced production due to service disruptions. In addition, social activities could be adversely affected by a range of factors, including isolation, mental health issues, and difficulties in accessing essential services. In addition, there may be adverse impacts on vulnerable populations who do not have access to health care or other support services. Policymakers should recognize that this approach could buy more time for preparation when health system capacity is tight, such as increasing medical and critical care storage and accelerating the vaccination rate for vulnerable populations.

As a transitional policy, we also simulated more extensive reopening scenarios involving no lockdowns and varying routine testing frequencies. We simulated what would happen if there were no more lockdowns. The results review that the outbreak will shock healthcare systems no matter what kind of reopening strategies. While implementing the 48-h routine test for all citizens could prevent an outbreak and control the extent of the epidemic, it is both labour-intensive and prohibitively expensive. It is also a challenge to track and track results effectively and guarantee the accuracy of test results. Additionally, long-term test exposure can make it difficult to accurately detect the presence of COVID-19, as it can take days or even weeks for the virus to become detectable in a person. So once the policymakers decide to drop lockdown and other restrictions, based on simulations, the research recommends that policymakers pay close attention to the cascading consequences of severe infections and possible Long-COVID effects. For example, public health systems should prepare for a potential long-term COVID-19 outbreak after reopening by implementing measures such as increased monitoring and tracking, public health education campaigns, and having medical professionals on standby. Additionally, they should ensure that healthcare systems are equipped to provide adequate care and support for those suffering from long-term symptoms of COVID-19, including access to mental health resources. Future work on the current research is to quantify the policy cost and discuss the policy optimisation problems. Also, the long COVID conditions and reinfections might cause a lack of labours in the market and employment. In future research, it is meaningful to quantitively estimate the corresponding damage caused by long COVID sequela and extra burdens on the public healthcare systems.

To prepare for potential labour shortages, employers should ensure their employees' health and safety. This can include providing access to adequate medical care and mental health resources for those suffering from the long-term effects of the virus or implementing flexible work arrangements that allow employees to work remotely. Additionally, employers should consider diversifying their workforce to include those who may not be able to work a traditional 9–5 schedule, such as senior citizens, disabled individuals, and those with caregiving responsibilities. As for mental health care, it is important to have access to mental health resources and support. This can include counselling services, peer support groups, and online resources. Additionally, employers should consider implementing flexible work arrangements that allow employees to work remotely if needed or provide time off to accommodate any mental health challenges they may face. It is also vital to create a supportive, understanding environment in the workplace to ensure people feel safe and supported. Finally, since reinfections correlate with death rates and severe symptoms, we suggest that people continue to follow public health guidelines, such as E.g. wearing masks, washing hands frequently and avoiding close contact with others whenever possible. In addition, it is important to be aware of any symptoms of reinfection and to consult a doctor if necessary. Getting vaccinated whenever possible is also recommended, as it can help reduce the risk and severity of reinfection.

## Methodology

**Figure 6:**
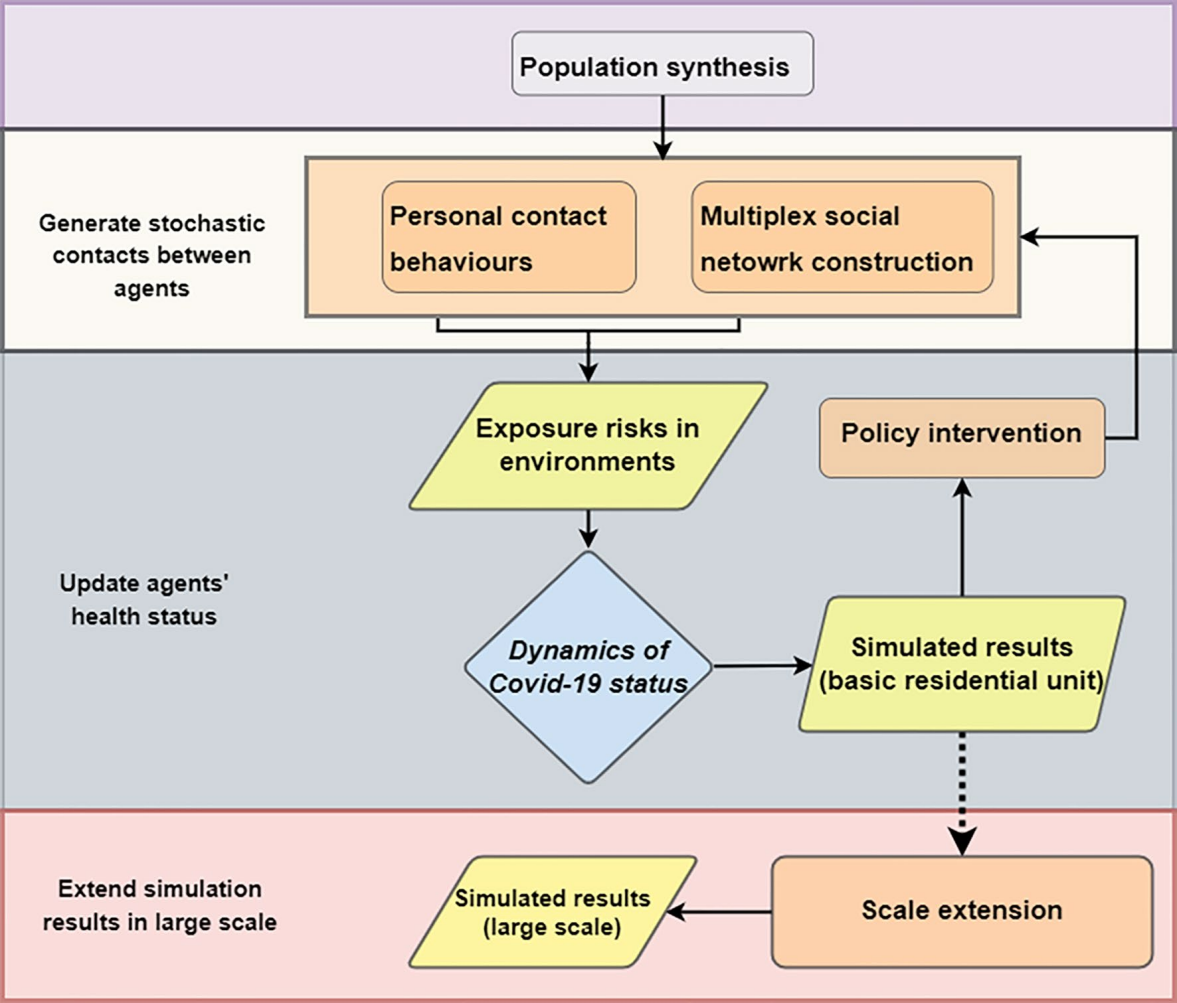
The simulation framework.

The current study utilized the fine-grained agent-based model to simulate how the virus spread through the dynamic contact network structures in three activity layers: household, residential community, and workplaces. The simulation has four steps by modules: population synthesis, simulation of the stochastic contacts by social networks, updates of the agent’s health status, and extension of simulation results shown in Fig. 6.

The agents are heterogeneous due to individual attributes, including age, occupation, social relationships etc. However, it is hard to find the entire datasets containing specific personal characteristics for some practical problems due to privacy or cost constraints ^13^.

Hence, firstly, population synthesis helps generate a disaggregate representation of the agents, which could match criteria like correlation structure and marginal sums. Following Truszkowska ^14^, the research collects multiple aggregated data from available census datasets and reports to synthesise the pseudo-population datasets displayed in Table 1. Each agent in CovFSA is assigned a unique identity as ${a}_{i}$. Also, since the age is a key factor correlated with not only contact and mobility activity^15,16^ but also the mortality rate and infection rate of COVID-19^17,18^, the current research distributes the age group to agents. Also, the agents have household identities following the empirical household size distribution. The occupation type and workplace information are collected from the local labour information at the aggregated level divided by age. Hence, the agents have corresponding occupation types in the conditional probability of age group and workplaces in conditional occupation types. The complete notations for the agent’s attributes are summarised in Table 1.

**Table 1:** The table for model notations.

Notation	Description
${\mathrm{a}}_{\rm{i}}$	The agent ${\mathrm{a}}_{\rm{i}}$, $\mathrm{i}$ is the identity
${\mathrm{a}}_{\rm{i, age}}$	$\mathrm{The agent } \, {\mathrm{a}}_{\rm{i}}$’s age group, an ordered categorical value
${\mathrm{a}}_{\rm{i, occ}}$	$\mathrm{The agent } \, {\mathrm{a}}_{\rm{i}}$’s occupation, a categorical value
${\mathrm{a}}_{\rm{i, Hid}}$	$\mathrm{The agent } \, {\mathrm{a}}_{\rm{i}}$’s household identity
${\mathrm{a}}_{\rm{i, Wid}}$	$\mathrm{The agent } \, {\mathrm{a}}_{\rm{i}}$’s workplace identity
${\mathrm{a}}_{\rm{i, Cid}}$	$\mathrm{The agent } \, {\mathrm{a}}_{\rm{i}}$’s community identity
${\mathrm{a}}_{\rm{i, t, h}}$	The agent ${\mathrm{a}}_{\rm{i}}{^{\prime}}\mathrm{s}$ health status on day $\mathrm{t},$ a categorical value
${\mathrm{a}}_{\rm{i, t, m}}$	The agent ${\mathrm{a}}_{\rm{i}}{^{\prime}}\mathrm{s}$ mobility status on day $\mathrm{t}, {\mathrm{a}}_{\rm{i, m, t}}\in ({0,1})$
${\mathrm{a}}_{\rm{i, vc}}$	The agent ${\mathrm{a}}_{\rm{i}}{^{\prime}}{\rm{s}}$ vaccine status, an ordered categorical value
${\mathrm{a}}_{\rm{i, crh}}$	The agent ${\mathrm{a}}_{\rm{i}}$’s contact rate in the household ${\mathrm{a}}_{\rm{i, crh}}\in {\mathrm{R}}^{+}.$
${\mathrm{a}}_{\rm{i, crw}}$	The agent ${\mathrm{a}}_{\rm{i}}$’s contact rate in the workplace ${\mathrm{a}}_{\rm{i, crw}}\in {\mathrm{R}}^{+}.$
${\mathrm{a}}_{\rm{i, crc}}$	The agent ${\mathrm{a}}_{\rm{i}}$’s contact rate in the community ${\mathrm{a}}_{\rm{i, crc}}\in {\mathrm{R}}^{+}.$
${\mathrm{CR}}_{\rm{Household}}$	The average level of contact rate in the household
${\mathrm{CR}}_{\rm{Occupation}}$	The average level of contact rate for a specific occupation
${\mathrm{CR}}_{\rm{Community}}$	The average level of contact rate in the community
$\mathrm{I}({\mathrm{a}}_{\rm{i, h}})$	The function to specify the Secondary Attack Rate(SAR) for each health status
$\uplambda ({\mathrm{a}}_{\rm{i, vc}})$	The function to specify the vaccine’s efficiency for COVID
${\mathrm{H}}_{\rm{i}}$	The household members of the agent ${\mathrm{a}}_{\rm{i}}$
${\mathrm{W}}_{\rm{i}}$	The colleagues of the agent ${\mathrm{a}}_{\rm{i}}$
${\mathrm{C}}_{\rm{i}}$	The community members of the agent ${\mathrm{a}}_{\rm{i}}$
${\mathrm{e}}_{\rm{i, t}}$	The exposure probability of the agent ${\mathrm{a}}_{\rm{i}}$ on day $\mathrm{t}$
$\upsigma$	The transition rate of the agent from exposed to infectious
${\mathrm{p}}_{\rm{asym}}$	The proportion of asymptomatically infectious cases among infected ones
${\mathrm{p}}_{\rm{severe}}$	The proportion of severe patients among symptomatically infectious cases
$\mathrm{d}$	The death rate of severe patients from severe patients
${\mathrm{r}}_{1},{\mathrm{r}}_{2},{\mathrm{r}}_{3}$	The recovery rates from asymptomatic (${\mathrm{r}}_{1}$), mild (${\mathrm{r}}_{2}$) and severe (${\mathrm{r}}_{3}$) patients to recover status
${\upepsilon }_{\rm{t}}$	The policy intervention’s efficiency, i.e. the probability of being detected and quarantined
${\upepsilon }_{\rm{max}}$	The maximum capacity of the government for policy intervention’s efficiency. ${\upepsilon }_{\rm{max}}\in ({0,1})$
$\upeta$	The threshold value for a lockdown of the community and its members
$\upupsilon$	The reaction speed of the local policy implementation
$\mathrm{D}{{\rm I}}_{\rm{t}}$	The set of detected infectious agents on day $\mathrm{t}$
${\rm T}$	The transmission probability matrix of health status for agent ${\mathrm{a}}_{\rm{i}}$ on day $\mathrm{t}$, whose element $\mathrm{\rm T}\left({\mathrm{a}}_{\rm{i, t, h}},{\mathrm{a}}_{\rm{i, t}+1,\mathrm{h}}\right)$ is the probability of status transmission

Then, based on the synthesised population information about the identity of households, workplaces (including schools) and communities, three different layers of networks by linking agents are constructed with the same household id, workplace id and community id. To illustrate the complex connections in the real world, we select 50 agents in two communities and display their multiplex social networks, as shown S1. Each agent is connected to his/her family in the household layer. And in the community layer, the agents have the dominant probability of connecting with their neighbours or community members. However, even though the agents in two communities are less likely to meet each other in a household or community, if they belong to the same workplace, the potential virus transmission chain can spread from one community to another through the contacts in the workplace layer.

The social contact networks for each agent ${a}_{i}$ is calculated by:

$$\forall i, {H}_{i}=\left\{{a}_{k} , \forall {a}_{k,Hid}={a}_{i,hid}\right\};{ W}_{i}=\left\{{a}_{k} , \forall {a}_{k,Wid}={a}_{i,Wid}\right\};{ C}_{i}=\left\{{a}_{k} , \forall {a}_{k,hid}={a}_{i,hid}\right\}$$

**Figure 7:**
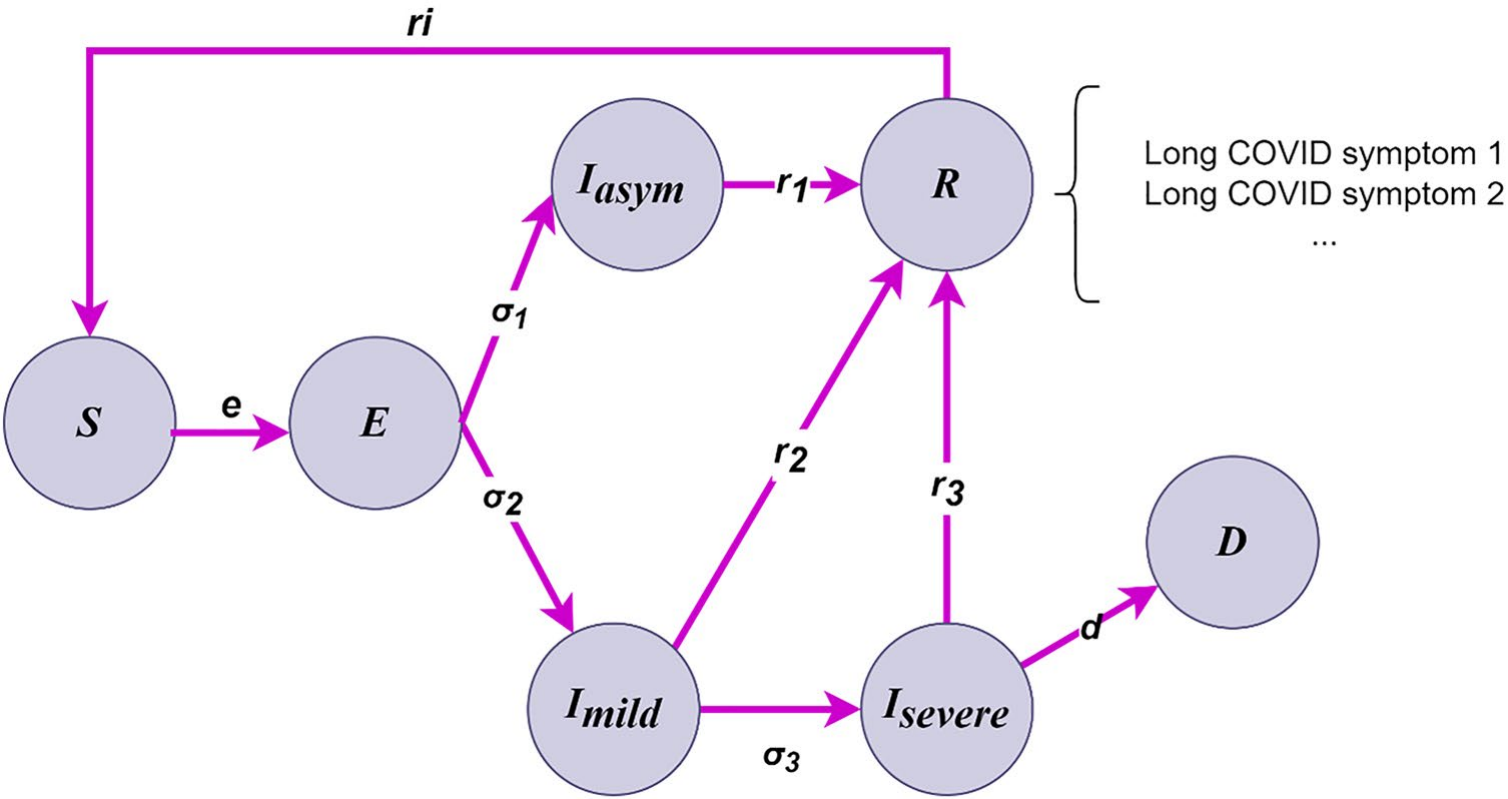
Flowchart of the transmission process of agent’s COVID health status.

The contact rates for agents in different layers are calculated by ${{\varvec{a}}}_{{\varvec{i}},{\varvec{c}}{\varvec{r}}{\varvec{l}}}=C{R}_{l}\boldsymbol{*}{\varvec{s}}{\varvec{a}}{\varvec{c}}{\varvec{l}}{\varvec{e}}({{\varvec{a}}}_{{\varvec{i}},{\varvec{a}}{\varvec{g}}{\varvec{e}}})$, where $scale({a}_{i,age})$ is the scale function for agent’s age group estimated from empirical study in China about the contact rates^15^ in S1. The mobility status describes the movement ability of an agent,$j$, on day $t$, denoted as ${{\varvec{a}}}_{{\varvec{j}},{\varvec{t}},{\varvec{m}}}$. We assume that the mobility of the agent will be influenced by personal health status ${a}_{health}$ and the policy intervention. If the agent is tested as COVID-19 positive and quarantined, the corresponding mobility status is 0. Then, the agent’s health status is updated by process shown in Fig. 7. An individual's health status is either susceptible, exposed, infectious, recovered or dead. The infectious individuals are further categorised as asymptomatic and symptomatic (mild and severe) according to their symptoms. Also, the recovered agents might become susceptible again due to the wanning effects of community^19^.

The transmission rate of each health status on day $t$ is heterogeneous depending on the environment and personal behaviours, especially for the exposure risk of susceptible agents.

${{\rm T}}_{i,t}\left({a}_{i,t,h},{a}_{i,t+1,h}\right)=P({a}_{i,t+1,h}{|a}_{i,t,h})$ is the probability of an agent ${a}_{i}$ on $t$ with health status, ${a}_{i,t,h}$, becoming ${a}_{i,t+1,h}$., where ${a}_{i,t+1,h}\in \{S,E,{I}_{asym},{I}_{mild},{I}_{severe},D,R\}$.

Assign the value in the health status transmission matrix for the agent ${a}_{i}$ on day $t$ by:

The exposed risk for susceptible agents:

$${\rm T}_{i,t}\left(S,E\right)= {e}_{i,t};$$

The transfer rate from exposed status to different infectious status:

$${\rm T}_{i,t}\left(E,{I}_{asym}\right)={\sigma }_{1}, {\rm T}_{i,t}\left(E,{I}_{mild}\right)={\sigma }_{2},{\rm T}_{i,t}\left({I}_{mild},{I}_{severe}\right)={\sigma }_{3};$$

The recovery rate from various infectious conditions to recovery:

$${\rm T}_{i,t}\left({I}_{asym},R\right)={r}_{1}, {\rm T}_{i,t}\left({I}_{mild},R\right)={r}_{2},{\rm T}_{i,t}\left({I}_{severe},R\right)={r}_{3}.$$

The death rate from severe infection to death:

$${\rm T}_{i,t}\left({I}_{severe},D\right)=d;$$

The reinfection rate from recovered agents being susceptible again:

$${\rm T}_{i,t}\left(R,S\right)=ri.$$

In addition, in this study, we select six major Long COVID symptoms discovered from the literature^19^ and use the estimated ratios to simulate the sequela conditions in the case study. The ratios of recovered agents for getting respiratory conditions is 3.655%, diseases of the nervous system is 2.644%, mental health burden is 2.888%, metabolic disorders is 3.008%, poor general well-being is 3.705%, and cardiovascular conditions 4.864%.

Then, the policy starts at the end of the simulated round by test the daily infected agents

$${\varvec{D}}{\boldsymbol{\rm I}}_{{\varvec{t}}}=\left\{{{\varvec{a}}}_{{\varvec{k}}} \right| {{\varvec{a}}}_{{\varvec{k}},{\varvec{t}},{\varvec{h}}}\in \{{{\varvec{I}}}_{{\varvec{a}}{\varvec{s}}{\varvec{y}}{\varvec{m}}},{{\varvec{I}}}_{{\varvec{s}}{\varvec{y}}{\varvec{m}}}\} \}\cap \{{{\varvec{a}}}_{{\varvec{k}}}\left| {{\varvec{a}}}_{{\varvec{k}},{\varvec{t}},{\varvec{m}}}>0 \right\}.$$

And the dynamic efficiency for policy intervention is set as ${{\varvec{\epsilon}}}_{{\varvec{t}}}=\frac{{{\varvec{\epsilon}}}_{{\varvec{m}}{\varvec{a}}{\varvec{x}}}}{1+{{\varvec{e}}}^{-{\varvec{v}} \, {\vert} {\varvec{D}}{{\varvec{I}}}_{{\varvec{t}}}|}}$***.***

Then for detected agents and their contacted agents, the mobility will be restricted to 0.

$${{\varvec{a}}}_{{\varvec{k}},{\varvec{m}},{\varvec{t}}}=0\,\,{\varvec{w}}.{\varvec{p}}.\,\,{{\varvec{\epsilon}}}_{{\varvec{t}}}\,,\,\,\forall \,{{\varvec{a}}}_{{\varvec{k}}}\in {\varvec{D}}{\boldsymbol{\rm I}}_{{\varvec{t}}}\cup \left\{{{\varvec{a}}}_{{\varvec{k}}}\right|\,{\forall \,{\varvec{a}}}_{{\varvec{j}}}\in {\varvec{D}}{{\varvec{I}}}_{{\varvec{t}}}\,,{{\varvec{a}}}_{{\varvec{k}}}\in {{\varvec{H}}}_{{\varvec{j}}}\cup {{\varvec{W}}}_{{\varvec{j}}}\}.$$

If the number of new infections exceeds the threshold values for lockdown, then all residential community members are restricted.

The following Algorithm 1 is the compact pseudo-code of complete simulation process.
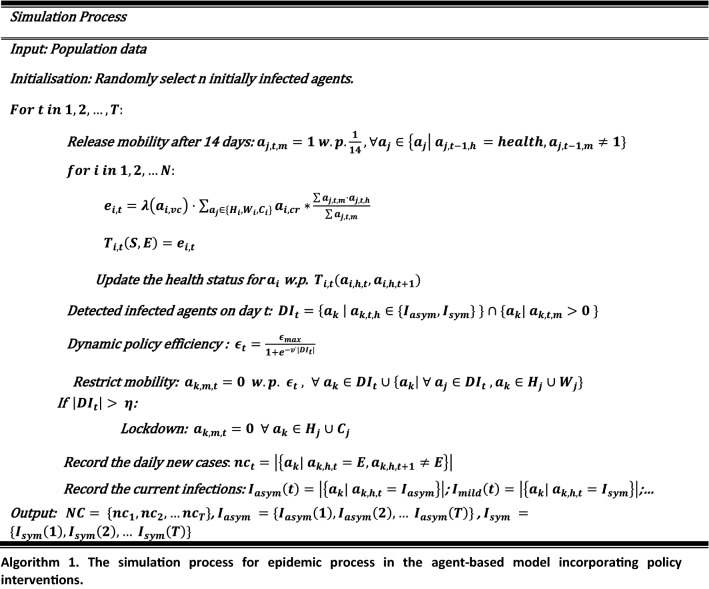


At last, the simulation results, which are the outputs of the above process, could be extended into larger regions. For example, the algorithm simulates the residential community-level results, and we employ the extension algorithms based on the distance^20^ and population to rescale the simulation results introduced in S2.

## Supplementary Information

Supplementary Information.

## Data Availability

All codes for agent-based model as well as its extension algorithms as well as simulation results are available for flexible users and shared in the https://github.com/seeingwu/CovFSA. The references for some open Cencus data in Shenzhen used in the current research are also listed in the README.md. No sensitive human data is collected and analysed in the research.
