# Supplementary material for: Modelling the reopen strategy from dynamic zero-COVID in China considering the sequela and reinfection
Source: Sci Rep. 2023 May 5;13:7343. doi: 10.1038/s41598-023-34207-7 (PMC10161982; doi:10.1038/s41598-023-34207-7)
Supplement: Supplementary file 1 — Supplementary Information. [file 41598_2023_34207_MOESM1_ESM.docx]

Supplementary information

This document is supplementary to the manuscript, including the complete descriptions of fine-agent-based models in S1 and S1. And the parameters about the case study in Shenzhen are presented in S3.

# S1 The fine-grained agent-based model for COVID simulation

In this section, the fine-grained agent-based model for COVID simulation (CovFSA) is introduced by dividing it into functional modules. The complete flowchart is illustrated in **Figure 1**. It consists of four parts: population synthesis, stochastic contact behaviours generation, health status update and simulation results extension. The clue of simulation logic follows the natural principles. First, the agents are assigned with personal demographic attributes by population synthesis supported by real-world data. Then, agents’ contact behaviours are heterogeneous due to their social roles in different organisations associated with individual characteristics generated through population synthesis. In addition, the agents’ exposure risks are stochastically determined by the contact activities between agents for updating their health status. Finally, the extension algorithm could extend the simulation results from the residential unit, which has around 30000 citizens in Shenzhen, to higher administrative divisions like subdistricts, districts or even the whole city.


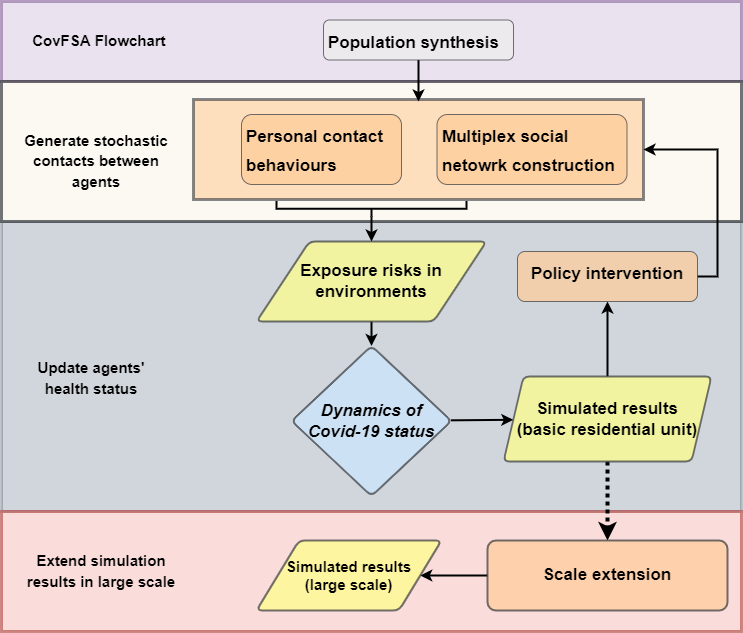


**Figure 1. The simulation framework.**

The subsequent subsections are constructed as follows. Section 1.1 introduces the population synthesis. Based on the agents generated in section 1.1, the social connections in multiplex networks and heterogeneous contact rates are stated in section 1.1. Then, the specific details of how to update Covid-19 status are demonstrated in section 1.3, before the policy intervention in section 1.4. Finally, section 1.5 displays the novel extension algorithm for fast simulating on a larger scaled population.

1.1 Population synthesis

In CovFSA, the agents are heterogeneous due to individual attributes, including age, occupation, social relationships etc. However, it is hard to find the entire datasets containing specific personal characteristics for some practical problems due to privacy or cost constraints ^1^. Hence, population synthesis helps generate a disaggregate representation of the agents, which could match criteria like correlation structure and marginal sums.

In CovFSA, following Truszkowska ^2^, the research collects multiple aggregated data from available census datasets and reports to synthesise the pseudo population datasets displayed in **Table 1**. Each agent in CovFSA is assigned a unique identity as $a_{i}$. Also, since the age is a key factor correlated with not only contact and mobility activity^3,4^ but also the mortality rate and infection rate of Covid-19^5,6^, the current research distributes the age group to agents. Also, the agents have the household identities following the empirical household size distribution. The occupation type and workplace information are collected from the local labour information at the aggregated level divided by age. Hence, the agents have corresponding occupation types in the conditional probability of age group and workplaces in conditional occupation types. In addition, the agents’ health status is another attribute for recording the trends of Covid during the simulation, which will be particularly introduced in section 1.3.

**Table 1. Basic personal attributes of an agent.**

| Notation | Description |
| --- | --- |
| $a_{i}$ | The agent $a_{i}$, $i$ is the identity. |
| $a_{i,age}$ | $The agent a_{i}$’s age group, an ordered categorical value. |
| $a_{i,occ}$ | $The agent a_{i}$’s occupation, a categorical value. |
| $a_{i,Hid}$ | $The agent a_{i}$’s household identity. |
| $a_{i,Wid}$ | $The agent a_{i}$’s workplace identity. |
| $a_{i,Cid}$ | $The agent a_{i}$’s community identity. |
| $a_{i,t,h}$ | The agent $a_{i}'s$ health status on day $t,$ a categorical value. |
| $a_{i,t,m}$ | The agent $a_{i}'s$ mobility status on day $t, a_{i,m,t}\in(0,1)$ |
| $a_{i,vc}$ | The agent $a_{i}'s$ vaccine status, an ordered categorical value. |
| $a_{i,crh}$ | The agent $a_{i}$’s contact rate in the household. $a_{i,crh}\in R^{+}.$ |
| $a_{i,crw}$ | The agent $a_{i}$’s contact rate in the workplace. $a_{i,crw}\in R^{+}.$ |
| $a_{i,crc}$ | The agent $a_{i}$’s contact rate in the community. $a_{i,crc}\in R^{+}.$ |
| $CR_{Household}$ | The average level of contact rate in the household. |
| $CR_{Occupation}$ | The average level of contact rate for a specific occupation. |
| $CR_{Community}$ | The average level of contact rate in the community. |
| $I(a_{i,h})$ | The function to specify the Secondary Attack Rate(SAR) for each health status. |
| $\lambda(a_{i,vc})$ | The function to specify the vaccine’s efficiency for COVID. |
| $H_{i}$ | The household members of the agent $a_{i}$. |
| $W_{i}$ | The colleagues of the agent $a_{i}$. |
| $C_{i}$ | The community members of the agent $a_{i}$. |
| $e_{i,t}$ | The exposure probability of the agent $a_{i}$ on day$t$. |
| $\sigma$ | The transition rate of the agent from exposed to infectious. |
| $p_{asym}$ | The proportion of asymptomatically infectious cases among infected ones. |
| $p_{severe}$ | The proportion of severe patients among symptomatically infectious cases. |
| $d$ | The death rate of severe patients from severe patients. |
| $r_{1},r_{2},r_{3}$ | The recovery rates from asymptomatic ($r_{1}$), mild ($r_{2}$) and severe ($r_{3}$) patients to recover status. |
| $\epsilon_{t}$ | The policy intervention’s efficiency, i.e. the probability of being detected and quarantined. |
| $\epsilon_{max}$ | The maximum capacity of the government for policy intervention’s efficiency. $\epsilon_{max}\in(0,1)$ |
| $\eta$ | The threshold value for a lockdown of the community and its members. |
| $\upsilon$ | The reaction speed of the local policy implementation. |
| $DI_{t}$ | The set of detected infectious agents on day $t$. |
| $T$ | The transmission probability matrix of health status for agent $a_{i}$ on day $t$, whose element $T\left( a_{i,t,h},a_{i,t+1,h} \right)$ is the probability of status transmission. |

1.2 Stochastic contacts in multiplex social networks

1.2.1 Generate multiplex social connections

In CovFSA, we approximate the real-life scenario by separating people’s main social activities into discrete layers of contact networks. Based on the synthesised population information about the identity of households, workplaces (including schools) and communities, three different layers of networks by linking agents are constructed with the same household id, workplace id and community id. To illustrate the complex connections in real word, we select 50 agents in two communities and display the multiplex social networks of them as shown in **Figure 2**. In household layer, each agent is connected within his/her family members. And in the community layer, the agents have dominant probability connect with their neighbours or community members. However, even though the agents in two communities are less likely to meet each other in household or community, if they belong to the same workplace, the potential virus transmission chain can spread from one community to another community through the contacts in the workplace layer.

By searching all agents’ id and their household id, workplace id and community id, the nodes within each household, workplace and community are collected and determined. We denote that $H=\{H_{i}, i=1,\ldots, h_{n}\}$, $W={\{W}_{i}, i=1,2,\ldots, w_{n}\}$ and $C=\{C_{i}, i=1,2,\ldots, c_{n}\}$ are sets of households, workplaces and communities, respectively. And $H_{i}=\{a_{j}|a_{household}=i\}$, $W_{i}=\{a_{j}|a_{workplace}=i\}$, $C_{i}\in=\{a_{j}|a_{community}=i\}$, are subsets of agents who share the same household, workplace or community. The extracted social relationships from population information are used to compute the exposure risks in each layer, which will be specifically descripted in section 1.3.


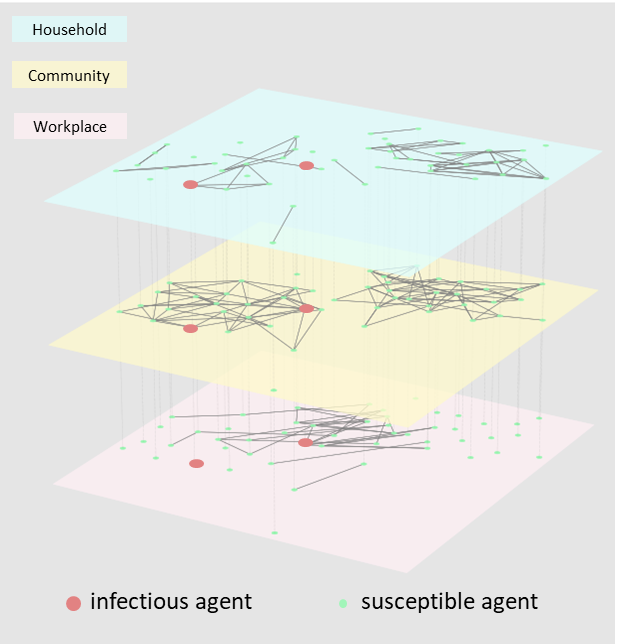


**Figure 2. The snapshot of multiplex networks consisting of 50 agents in three layers. The red points are infectious agents and green ones are susceptible. Edges between agents mean the agents know each other in the social organisation and might have physical contacts.**

1.2.2 Assign the agents’ stochastic contacts

Before beginning the simulation, it is essential to define individual contact behaviours, which affect the epidemic trend in vital. The potential infected cases (i.e. agents contact with the infectious ones) are determined based on the social connections described in section 1.1.1. And agents’ contact rates are assigned by heterogeneous age and location, denoted as $cr_{age, layer}.$ Here the current research assumes the contact rates are defined as the number of close physical contacts between individuals per day. For community and household layers, the agent $a$’s contact rate with other agents is the product of a mean value in community(household) with the age scale. Taking the household contact rate as an example, the contact rates in household of agent $a$ is

$$cr_{household}(a)=cr_{household}*scale(a_{age}),$$

where a concrete function of age scaling is $scale\left( x \right)=\left\{ \begin{aligned} 1.175, x=1 \\ 1, x=2 \\ 0.625, x=3,4 \end{aligned} \right.$and the scale ratio is estimated from empirical study in China about the contact rates ^3^.

The research also considers the heterogeneous occupation types to disaggregate the contact rates in the workplace layer through

$$cr_{workplace}(a)=cr_{occupation}*scale(a_{age}).$$

The mobility status describes the movement ability of an agent, denoted as $a_{m}$. We assume that the mobility of the agent will be influenced by personal health status $a_{health}$ and the policy intervention. If the agent is tested as COVID-19 positive and quarantined, the corresponding mobility status is 0. The discrete function of mobility influenced by health is $M\left( x \right), x\in\left\{ S,E,I_{asym},I_{sym},ICU,R,D \right\}$ and takes values in Table 2.

**Table 2. Assumed mobility as a function of the agent’s health status** $\boldsymbol{M(}\boldsymbol{a}_{\boldsymbol{health}}\boldsymbol{)}$**.**

| $a_{health}$ | $mobility$ |
| --- | --- |
| $S,E,I\_asym$*, R* | $1$ |
| $I\_sym$ | $0.6$ |
| $ICU,D$ | $0$ |

1.3 The dynamics of agent’s health status

A schematic diagram of the model structure used in CovFSA is shown in **Figure 3**. An individual's health status is either susceptible, exposed, infectious, recovered or dead. The infectious individuals are further categorised as asymptomatic and symptomatic (mild and severe) according to their symptoms.


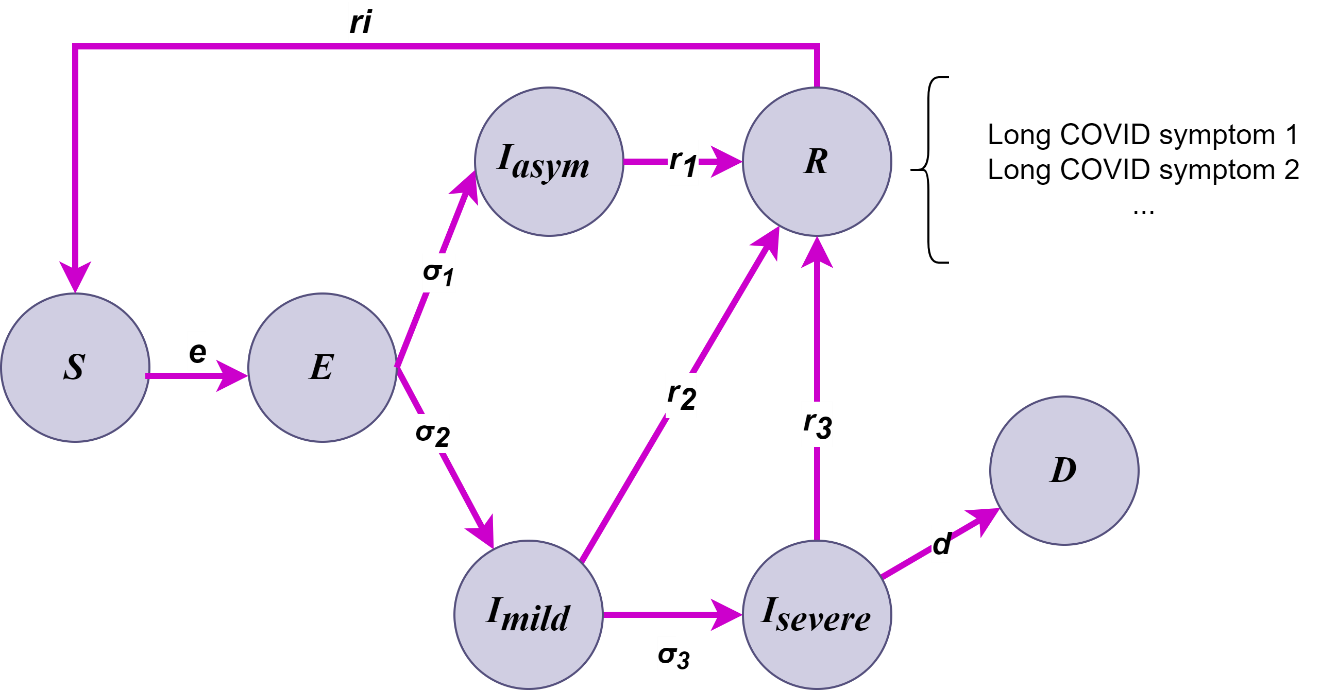


**Figure 3. The flowchart of health status in the agent-based model.**

The transmission rate of each health status on day $t$ is heterogeneous depending on the environment and personal behaviours, especially for the exposure risk of susceptible agents.

$T_{i,t}\left( a_{i,t,h},a_{i,t+1,h} \right)=P(a_{i,t+1,h}{|a}_{i,t,h})$ is the probability of an agent $a_{i}$ on $t$ with health status, $a_{i,t,h}$, becoming $a_{i,t+1,h}$., where $a_{i,t+1,h}\in\{S,E,I_{asym},I_{mild},I_{severe},D,R\}$.

Assign the value in the health status transmission matrix for the agent $a_{i}$ on day $t$ by:

The exposed risk for susceptible agents:

$T_{i,t}\left( S,E \right)= e_{i,t}$;

The transfer rate from exposed status to different infectious status:

$$T_{i,t}\left( E,I_{asym} \right)=\sigma_{1}, T_{i,t}\left( E,I_{mild} \right)=\sigma_{2},T_{i,t}\left( I_{mild},I_{severe} \right)=\sigma_{3};$$

The recovery rate from various infectious conditions to recovery:

$$T_{i,t}\left( I_{asym},R \right)=r_{1}, T_{i,t}\left( I_{mild},R \right)=r_{2},T_{i,t}\left( I_{severe},R \right)=r_{3};$$

The death rate from severe infection to death:

$T_{i,t}\left( I_{severe},D \right)=d$*;*

The reinfection rate from recovered agents being susceptible again:

$T_{i,t}\left( R,S \right)=ri$*;*

Unlike the traditional SEIR model, CovFSA updates an agent’s health status by considering the individual contacts with possible infectious agents daily. The susceptible agents will become exposed in probability if they share the same layers with infectious agents who are activate (i.e. $a_{m}>0$).

The secondary attack rate (SAR) is the probability that an infectious person infects a susceptible person through close contact during the infector’s whole infectious period ^4^. The current model also considers the heterogeneous infectiousness of different infection periods and uses the following SAR estimates in the model, $I\left( a_{health} \right).$ Then, the spatial-temporal exposure risk is estimated based on the mobility weighted sum of infectiousness in each household, workplace and community.

For susceptible agents, $a_{health}=S$, their exposure risk in the household, workplace and community is related to the number of infectious agents in the same environments and the personal contact rates.

where $H_{i}$is the set of agents that are household members of the agent$a_{i}$.

Then, the total exposure risk on that day, $\mathcal{e}$, of a susceptible agent is the time-weighted sum of each social network layer.

$\sum_{{\boldsymbol{a}_{\boldsymbol{j}}\boldsymbol{\in\{H}}_{\boldsymbol{i}}\boldsymbol{,}\boldsymbol{W}_{\boldsymbol{i}}\boldsymbol{,}\boldsymbol{C}_{\boldsymbol{i}}\boldsymbol{\}}} \boldsymbol{a}_{\boldsymbol{i,cr}}\boldsymbol{*}\frac{\sum\boldsymbol{a}_{\boldsymbol{j,t,m}}\boldsymbol{\cdot}\boldsymbol{a}_{\boldsymbol{j,t,h}}}{\sum\boldsymbol{a}_{\boldsymbol{j,t,m}}}$

where $\lambda(a_{vc})$ is the protection rate caused by the vaccine, and the number of vaccine doses is assigned by empirical distribution in each agent’s age group, denoted as $a_{vc}\in[1,2,3]$. where we use an approximated linear function of vaccine doses

$$\lambda(a_{vc})=1-0.3a_{vc},$$

and $l\in[household, community, workplace]$ represents the layers of multiplex network, $w_{l}$ is the time weight spent on the layer $l$, $w_{l}\in[0,1]$.

The COVID-19 status of each agent is iteratively updated according to the environment's exposure risk and contact behaviours. The update procedure is summarised in the following pseudocode.

1.4 Policy intervention: The Dynamic COVID-Zero Policy

The Dynamic COVID-Zero (DCZ) consistently adopted in China includes regular screening and quarantine in the city^7^. The DCZ expects to control the virus spread by accurately restricting the mobility of potential infections. Specifically, the local government regularly carries the routine test for every citizen, and if there are newly found cases, both themselves and their spatiotemporal accompanies are quarantined. Furthermore, if the decision-makers think the situation is out of control, the lockdown will be implemented. To reproduce the epidemic in recent waves and evaluate the current policy, it is essential to mimic the actual DCZ’s implementation in China. However, there are many realistic constraints in executing the policy. First, the test capacity and policy compliance may limit regular screening on a large scale. Second, due to the privacy issue and the efficiency of track and trace, information on close contacts of infected patients is missed. Hence, to quantitively embed the DCZ into CovFSA, the policy efficiency is dynamically changing and is related to the daily cases and policy capacity.

First, the newly infected agents at day $t$ are in the set of agents who are infected and not detected before, denoted as

$\boldsymbol{D}\boldsymbol{I}_{\boldsymbol{t}}\boldsymbol{=}\left\{ \boldsymbol{a}_{\boldsymbol{k}} \right| \boldsymbol{a}_{\boldsymbol{k,t,h}}\boldsymbol{\in\{}\boldsymbol{I}_{\boldsymbol{asym}}\boldsymbol{,}\boldsymbol{I}_{\boldsymbol{sym}}\boldsymbol{\} \}\cap\{}\boldsymbol{a}_{\boldsymbol{k}}\boldsymbol{|}\boldsymbol{a}_{\boldsymbol{k,t,m}}\boldsymbol{>0 \}}$

Then, the policy’s dynamic efficiency on day $t$, $\boldsymbol{\epsilon}_{\boldsymbol{t}}$, is assumed and mapped by maximum screen capacity $\boldsymbol{\epsilon}_{\boldsymbol{max}}$, policy reaction speed $v$ , and $|I_{new}(t-1)$| in a logistic function:

$\boldsymbol{\epsilon}_{\boldsymbol{t}}\boldsymbol{=}\frac{\boldsymbol{\epsilon}_{\boldsymbol{max}}}{\boldsymbol{1+}\boldsymbol{e}^{\boldsymbol{-v ̇|D}\boldsymbol{I}_{\boldsymbol{t}}\boldsymbol{|}}}$.

The $\boldsymbol{\epsilon}_{\boldsymbol{max}}$ is the maximum value of daily test capacity ranging from zero to one. Another shape parameter in logistic function $v,v\geq0$ represents the dynamic reaction speed in which the government activates the emergent actions to search for potential infections once newly infected agents are found. Once the infectious agents are detected, they and those who share the same social groups are restricted. For detected agents, they will be isolated by setting $w_{l}$ = 0 for any layer. As for agents who share the same households and workplaces, they will be quarantined by setting $w_{household}$ and $w_{workplace}$ = 0 or not in a dynamic probability $\boldsymbol{\epsilon}_{\boldsymbol{t}}$. As for agents in the same community, the research follows the Shenzhen CDC’s local lockdown rule. By setting a threshold value, that if the number of infections in the community are larger than the threshold, the agents within the community are restricted by setting $w_{community}$ and $w_{workplace}$ = 0 or not in a dynamic probability $\boldsymbol{\epsilon}_{\boldsymbol{t}}$.

In addition, the newly infected agents are tested positive in a binary random variable with probability as $\boldsymbol{\epsilon}_{\boldsymbol{t}}$, which means if $a\in\boldsymbol{D}\boldsymbol{I}_{\boldsymbol{t}}$ and $a$ is detected successfully by screening in a probability $\boldsymbol{\epsilon}_{\boldsymbol{t}}$. According to the published documents about DCZ, the set of all detected agents and their household members, colleges and community members are restricted to different levels. For example, the positive agents ($a_{health}=I_{asym} or I_{sym}$) and their household members are isolated by setting mobility to 0. The community members and colleagues are required to stay at home for at least three days. Therefore, the mobility weight in the community and workplace is set as zero at that period, which means they are not allowed to go out. The illustration of DCZ on social connections is in **Figure 4**.

**
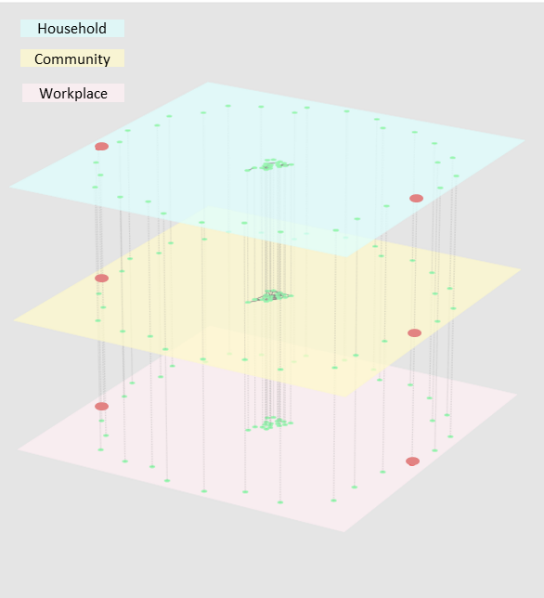

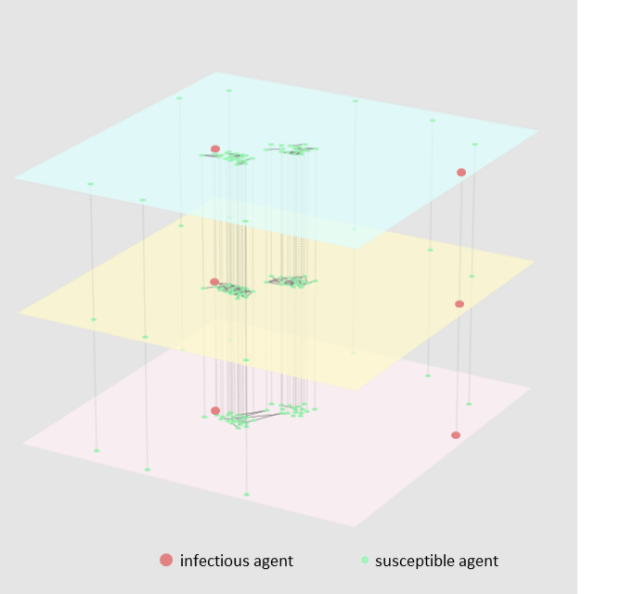
**

**Figure 4. The snapshots of multiplex networks consist of 50 agents in three layers under two scenarios. The left figure shows the social connection under 90% policy efficiency. Two infected agents and their accompanies are quarantined. The right figure is under 50% policy efficiency. Only one of the infected agents is detected, and partial accompanies are quarantined.**

In addition, the local lockdown in the risk region is the important intervention. The CovFSA simulates the lockdown by importing the threshold parameter,$\eta$. If the number of detected cases is larger than $\eta$, then all community members are restricted by setting $w_{l}=0$.

# S2 Time-delayed Geodemographic scaling

One significant challenge with ABM is that it requires cumbersome memory or processor usage levels ^8^ when simulating a vast number of agents to capture large-scale epidemics. Consequently, many ABMs utilised an optional scaling factor where a single agent in the model is assumed to represent multiple people in the real world. However, the simple scale factor may reduce the ABM’s heterogeneity. To circumvent this problem, a novel scaling method is proposed to simulate the pandemic in a large-scale population through the real geo-information. Considering the real scenario of initial Covid-19 outbreak, the patient zero first spreads his/her neighbourhood and workplaces in the residential community level, then epidemics could extend to the larger administrative level, such as sub-district, districts and cities. Hence, the current research proposed a novel method to extend the simulated results from small size to larger administrative levels without simulating the whole population directly to save the computational source. The ideology is that the CovFSA would simulate multiple rounds of epidemics in small size independently. Because of the stochasticity of agents’ behaviours and initialisation conditions, the simulated results would perform divergence, which reflects the unmeasured uncertainty in reality. Then, considering the heterogeneous geodemographic information, the combination of selected simulation results in a certain rule is utilised to simulate the epidemics in a larger scale, which will be described in detail as following.

In CovFSA, the community with 30000 citizens, the average size of citizens in a residential community estimated by sixth census in Shenzhen^[[1]](#footnote-1)^, acts as a basic unit. A simulated sequence in $i^{th}$ experiment, $\mathcal{S}_{i}$, represents the simulated daily infections of the community in T days, $\mathcal{S}_{i}=\left[ \mathcal{S}_{i0},\mathcal{S}_{i1}, \ldots, \mathcal{S}_{iT} \right], \mathcal{S}_{ij}\in\mathbb{Z}^{+}$.

If there are $k$ residential communities in the sub-district with population ${Pop}_{i},i=1,2,\ldots,k$, and $m (m>k)$ duplicate simulated results at the unit level independently, storing in a set $\mathcal{M=\{}\mathcal{S}_{1},\mathcal{S}_{2}, \ldots,\mathcal{S}_{m}\}$, for generating the robust simulation in sub-district level, we rearrange $k$ elements of the set $\mathcal{M}$ without repetitions to form an ordered sequence forming a partial permutation set, $\mathbb{P}$. The number of elements in $\mathbb{P}$ is $\frac{n!}{\left( n-k \right)!}$. One arbitrary permutation, $\rho_{i}$ , of $M$ with $k$ sequences could be represented as $\rho_{i}= \left( \mathcal{S}_{i1}, \mathcal{S}_{i2}, \ldots, \mathcal{S}_{ik} \right)\mathbb{\in P,}$where $\mathcal{S}_{ij}$ is selected randomly from M and $j$ is the order of this sequence.

We assume that residential communities’ population size is proportional to the epidemic results and are tiled in hexagons approximately where the distance between them can be measured by hexagonal degree. For example, if one residential community $c_{1}$ outbreaks first in a sub-district, the one of the estimated daily infection curves of the sub-district is

$$\Gamma(\rho_{i})=\sum_{j=1}^{k} \frac{Pop_{j}}{Pop_{1}}*S_{ij}(t-d(j)*\triangle\tau),$$

where $\frac{{Pop}_{j}}{{Pop}_{1}}$ is the proportional scale of population size for each residential community, $d\left( j \right)=max\{n\mathbb{\in Z|}n\leq\frac{j-1}{6}\}$ is the hexagonal distance measure between residential communities, and $\triangle\tau$ is the unit time-lag spreading to subsequent regions. By this approach, to generate robust simulation results in many communities and larger administrative authorities, it is time-friendly to get $\binom{m}{k}$experiments with only $m$ duplicating simulations.

From$\binom{m}{k}$ experiment results, we use the mean value representing the regional epidemic trends and applying the t-statistics to compute the confidence interval of simulation. Suppose we randomly collected $|L|$ experiment results, $|L|<\binom{m}{k}$, and the set of experimental sequences is $L=\{\Gamma(\rho_{1}),\Gamma(\rho_{2}),\ldots,\Gamma(\rho_{\left| L \right|})\}$. Then, the mean value and sample variance of extended simulation results, denoted as $\hat{\mu}$ and $\hat{\sigma^{2}}$ :

$$\widehat{\mu}\triangleq\frac{1}{|L|}\sum_{i} \Gamma(\rho_{i}),$$

$$\widehat{\sigma^{2}}\triangleq\frac{1}{|L|-1}\sum_{i} (\Gamma(\rho_{i})-\widehat{\mu})^{2}.$$

Therefore, the approximate 95% confidence interval of simulated epidemics in higher administrative level is represented as

$$[\widehat{\mu}-1.96\frac{\widehat{\sigma}}{\sqrt{|L|}},\widehat{\mu}+1.96\frac{\widehat{\sigma}}{\sqrt{|L|}}].$$

Finally, for simplifying the description with one equation replacing the above, the current research denotes the set of independent simulation results in lower administrative level as $\mathcal{M}_{l}$, and the procedure of time-delayed geodemographic scaling as $\phi$, to represent the extended simulations in higher administrative level as $\mathcal{M}_{h}=\phi(\mathcal{M}_{l})$.

# S3 Parameters

Shenzhen is a major sub-provincial city with 17.56 million citizens bordering Hong Kong. As one of the special economic zones of China, it acts as the global centre in technology, manufacturing, business, economics and transportation with the world’s fourth busiest container port. Due to its significant roles in economy and special geographic position, The Shenzhen government urges to find the balance between the threats from COVID-19 spread and potential effects on socio-economic activities caused by policy interventions. The regular city-level testing is implemented even after more than a month of clearance of infections in Shenzhen. In this study, CovFSA traces back the COVID-19 outbreak in Shenzhen, which started from February 2022 and ended in a month.

Since the pandemics usually spread from one small region to larger ones. Some residential communities happened outbreaks in previous cases, and the situations are controlled by stringent interventions at that time. Hence, the first part of case study section is to validate the CovFSA by using reported cases in Shenzhen. The second part would discuss what would happen if the government took different interventions at that time. The current research assumes that epidemics spread from the residential community to the whole city in Figure 7. The simulation results represent the epidemics from one residential community that initially occurs infected agents at first. Then, according to the time-delayed geodemographic scaling method in S2, the simulation results are processed and used for showing the pandemic trends in city level.

For enhancing the credibility of simulation results, the current case study adopts the local data from Shenzhen population census ^9^to generate the agents representing Shenzhen citizens. **Table 3** and **Table 4** show the individual contact rates divided by occupation before the lockdown referenced by the previous survey ^3^. The parameters about the local age-stratified vaccine rates are shown in **Table 5**. As for the critical parameters related to infectiousness of Omicron, the research goes through a comprehensive literature review and selects them in **Table 6** and **Table 7**.

**Table 3. Contact rates in the workplace layer.**

| Occupation type | $cr_{occupation}$ |
| --- | --- |
| *School student* | $U\sim(10,13)$ |
| *Unemployed* | $U\sim(3.6,9.4)$ |
| *Others* | $U\sim(8,10)$ |

**Table 4. Base contact rates in community and household.**

| Occupation type | $Contact rates$ |
| --- | --- |
| $cr_{household}$ | $7$ |
| $cr_{community}$ | $5$ |

**Table 5. The age-stratified vaccine cover rates reported in Shenzhen in March 2022.**

| Age group | Zero dose (%) | One dose (%) | Two doses (%) | Three doses (%) |
| --- | --- | --- | --- | --- |
| *1* | *30* | *50* | *20* | *0* |
| *2* | *1* | *41* | *38* | *20* |
| *3* | *4* | *39* | *36* | *21* |
| *4* | *15* | *38* | *33* | *14* |

It is worth noting that the ratio of symptomatic and asymptomatic cases is estimated by reported cases from Shenzhen CDC. Also, since there is no death case reported, the current research only considers the proportion of patients requiring the ICU.

**Table 6. The parameter tables for COVID-19 BA.2 dynamics**

| Parameter | Value | Literature |
| --- | --- | --- |
| $\sigma$ | 3.6 days ([2.3, 4.9] 95% CI) | ^10,11^. |
| $r_{1},r_{2}$ | 5(13.1) mean(std) | ^12^ |
| $r_{3}$ | 7.78 ([6.99,8.63] 95% CI) | ^13^ |
| $ri$ | 0.7(SD:0.33) per 10000 person -days | ^14^ |
| $d$ | N/A | The death cases caused by Omicron in Shenzhen are too small to estimate the evidential parameter. |
| $\sigma_{3}$ | 0.47% | ^15^ |

**Table 7. SAR as a function of the agent’s health status I(**$\boldsymbol{a}_{\boldsymbol{health}}$**).**

| $a_{health}$ | $SAR$ |
| --- | --- |
| $S$ | $0$ |
| $E$ | $U\sim(0.02,0.1)$ |
| $I\_sym$ | $U\sim(0.2,0.64)$ |
| $I\_asym$ | $U\sim(0.16,0.55)$ |
| $D$ | $U\sim(0.06,0.45)$ |
| $R$ | *0* |

By calibrating the hyper-parameters in initialisation seed and policy efficiency at that time, CovFSA could simulate the cumulative curves fitting the reported true values stated in section 3.1. Then, in section 3.2, the current study predicts the epidemics under various re-open strategies and discusses the reliability of prediction results. And the used parameters in this section are available in Appendix. The current version of CovFSA is coded by Python 3.7.3 and written in Jupyter Notebook. The execution time of a 180-day simulation with 30000 agents including policy interventions requires around 105s. The processor used in the simulation is Intel(R) Xeon(R) W-2123 CPU @ 3.60GHz.


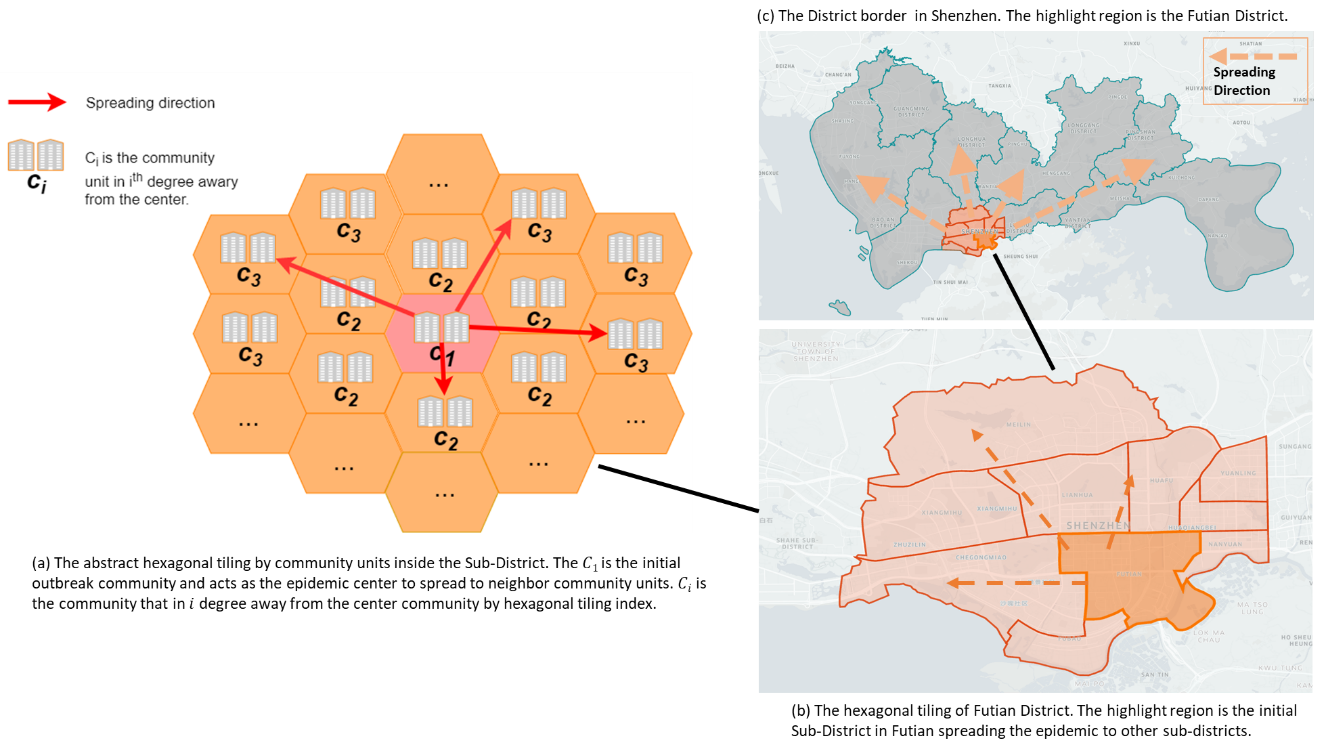


**Figure 5. The assumed epidemic spread path from community to the city.**

**Figure 5** displays the epidemic path from one residential community unit to the subdistrict level, the district and the city. In implementation on each scenario, the research first run simulations independently and save the residential community results as $\mathcal{M}_{c}=\{\mathcal{S}_{1},\mathcal{S}_{2}, \ldots,\mathcal{S}_{m}\}$. Denoting the Time-delayed Geodemographic scaling as function $\phi$, $\mathcal{M}_{subdistrict}=\phi(\mathcal{M}_{c})$ is the set of simulation results in subdistrict level just as shown in Figure 5. (a). Again, by repeating the similar process, the subdistrict results are extended to district level as $\mathcal{M}_{district}= \phi\circ\phi(\mathcal{M}_{c})$. And the district results finally compose the city-level simulations represented as $\mathcal{M}_{city}= \phi(\mathcal{M}_{district})$. All three scenarios discussed are city-level results, with $\triangle\tau$ in $\phi(\mathcal{M}_{c})$ as 0. In $\phi\left( \mathcal{M}_{subdistrict} \right) and \phi(\mathcal{M}_{district})= 3.6 ([2.3, 4.9] )$ as same as the incubation time shown in **Table 6**. $\frac{{Pop}_{i}}{{Pop}_{1}}$ in $\phi(\mathcal{M}_{c})$ and $\phi\left( \mathcal{M}_{subdistrict} \right)$ are assumed as 1 indicating that the population difference among communities and subdistricts are neglectable. For population scale in $\phi(\mathcal{M}_{district})$, the scaling vector is estimated through the real population data described in **Table 8**.

**Table 8. The population size in districts of Shenzhen from local census.**

| Index of district | 1 | 2 | 3 | 4 | 5 | 6 | 7 | 8 | 9 |
| --- | --- | --- | --- | --- | --- | --- | --- | --- | --- |
| $Pop_{i}$*(million)* | *1.55* | *1.79* | *2.52* | *3.97* | *1.14* | *1.09* | *4.47* | *0.55* | *0.21* |

# References

1. Müller, K. & Axhausen, K. W. Population synthesis for microsimulation: State of the art. 16.

2. Truszkowska, A. *et al.* High-Resolution Agent-Based Modeling of COVID-19 Spreading in a Small Town. *Adv. Theory Simul.* **4**, 2000277 (2021).

3. Zhang, J. & Litvinova, M. The impact of relaxing interventions on human contact patterns and SARS-CoV-2 transmission in China. *Sci. Adv.* 11 (2021).

4. Tsang, T. K. *et al.* Variability in transmission risk of SARS-CoV-2 in close contact settings: A contact tracing study in Shandong Province, China. *Epidemics* **39**, 100553 (2022).

5. CDC Weekly, C. & The Novel Coronavirus Pneumonia Emergency Response Epidemiology Team. The Epidemiological Characteristics of an Outbreak of 2019 Novel Coronavirus Diseases (COVID-19) — China, 2020. *China CDC Wkly.* **2**, 113–122 (2020).

6. Balabdaoui, F. & Mohr, D. Age-stratified model of the COVID-19 epidemic to analyze the impact of relaxing lockdown measures: nowcasting and forecasting for Switzerland. *medRxiv* 2020.05.08.20095059 (2020) doi:10.1101/2020.05.08.20095059.

7. Liu, J., Liu, M. & Liang, W. The Dynamic COVID-Zero Strategy in China. *China CDC Wkly.* **4**, 74–75 (2022).

8. Kerr, C. C. *et al.* Covasim: An agent-based model of COVID-19 dynamics and interventions. *PLOS Comput. Biol.* **17**, e1009149 (2021).

9. http://tjj.sz.gov.cn/ztzl/zt/szsdqcqgrkpc/.

10. Backer, J. A. *et al.* Shorter serial intervals in SARS-CoV-2 cases with Omicron BA.1 variant compared with Delta variant, the Netherlands, 13 to 26 December 2021. *Eurosurveillance* **27**, 2200042 (2022).

11. Del Águila-Mejía, J. *et al.* Secondary Attack Rate, Transmission and Incubation Periods, and Serial Interval of SARS-CoV-2 Omicron Variant, Spain. *Emerg. Infect. Dis.* **28**, 1224–1228 (2022).

12. Maslo, C. *et al.* Characteristics and Outcomes of Hospitalized Patients in South Africa During the COVID-19 Omicron Wave Compared With Previous Waves. *JAMA* **327**, 583 (2022).

13. Tan, E., Song, J., Deane, A. M. & Plummer, M. P. Global Impact of Coronavirus Disease 2019 Infection Requiring Admission to the ICU: A Systematic Review and Meta-analysis. *Chest* **159**, 524–536 (2021).

14. Deng, L. *et al.* Risk of SARS-CoV-2 reinfection: a systematic review and meta-analysis. *Sci. Rep.* **12**, 20763 (2022).

15. Wang, L. *et al.* COVID infection rates, clinical outcomes, and racial/ethnic and gender disparities before and after Omicron emerged in the US. *MedRxiv Prepr. Serv. Health Sci.* 2022.02.21.22271300 (2022) doi:10.1101/2022.02.21.22271300.

1. http://tjj.sz.gov.cn/zwgk/zfxxgkml/tjsj/tjgb/content/post_3084945.html [↑](#footnote-ref-1)
